# Intervening on Social Comparisons on Social Media: Electronic Daily Diary Pilot Study

**DOI:** 10.2196/42024

**Published:** 2023-04-28

**Authors:** Fernanda C Andrade, Savannah Erwin, Kaitlyn Burnell, Jalisa Jackson, Marley Storch, Julia Nicholas, Nancy Zucker

**Affiliations:** 1 Department of Psychology & Neuroscience Duke University Durham, NC United States; 2 Department of Psychology and Neuroscience University of North Carolina at Chapel Hill Chapel Hill, NC United States; 3 Department of Psychological and Brain Sciences University of Louisville Louisville, KY United States

**Keywords:** social media, social comparison, young adults, social savoring, intervention, self-esteem, depression

## Abstract

**Background:**

Literature has underscored the dark aspects of social media use, including associations with depressive symptoms, feelings of social isolation, and diminished self-esteem. Social comparison, the process of evaluating oneself relative to another person, is thought to contribute to these negative experiences such that people with a stronger tendency to compare themselves with others are particularly susceptible to the detrimental effects of social media. Social media as a form of social connection and communication is nevertheless an inevitable—and arguably integral—part of life, particularly for young adults. Therefore, there is a need to investigate strategies that could alter the manner in which people interact with social media to minimize its detrimental effects and maximize the feelings of affiliation and connection.

**Objective:**

This pilot study examined the feasibility, acceptability, and effectiveness of a brief web-based intervention designed to alter engagement with social media and promote psychological well-being by encouraging social savoring as an alternative to social comparison. Social savoring was operationalized as experiencing joyful emotions related to the happiness of someone else’s experiences (ie, feeling happy for someone else).

**Methods:**

Following an intensive longitudinal design, 55 college students (mean age 19.29, SD 0.93 years; n=43, 78% women and n=23, 42% White) completed baseline measures (individual differences, psychological well-being, connectedness, and social media use) and then 14 days of daily surveys on their social media activity and well-being. On day 8, the group that was randomized to receive the intervention watched a video instructing them on the skill of social savoring and was asked to practice this skill during days 8 to 14.

**Results:**

Overall, participants reported positive perceptions of the intervention. Participants who watched the intervention video reported significantly higher performance self-esteem (*P*=.02) at posttest than those in the control condition, after controlling for baseline levels. Participants also reported significantly higher state self-esteem (*P*=.01) on days in which they engaged in more social savoring while using social media, and the use of social savoring increased significantly (*P*=.01) over time, suggesting that participants found it helpful. Participants in both conditions reported significantly lower levels of social comparison (control: *P*=.01; intervention: *P*=.002) and higher levels of connectedness (control: *P*<.001; intervention: *P*=.001) at posttest than at baseline.

**Conclusions:**

Initial evidence from this pilot study suggests that a web-based social savoring intervention may help minimize the potentially harmful consequences of social media use, at least in some domains. Future work is needed to examine the effectiveness and acceptance of this intervention in different age groups and in clinical samples that are in part characterized by higher levels of comparison with others (eg, people with eating disorders).

## Introduction

### Social Comparisons on Social Media

Today, 72% of the American public uses some type of social media, and most people interact with at least 1 social media site daily [1]. Social media platforms help people synchronously and asynchronously connect with others. Adolescents and young adults, in particular, use social media to build, maintain, and strengthen their social networks [2-5]. Beyond fortifying social networks, engagement with social media content has been associated with a tendency to engage in social comparisons [6]. Social comparison, the act of comparing oneself with others, stems, in part, from people’s need to evaluate their own opinions and abilities by comparing themselves with other people [7]. Such comparisons can have positive intentions and outcomes, such as motivating self-improvement, enhancing learning, and fostering positive self-views [8].

Notwithstanding, exposure to social media content can increase the risk of poor mental health outcomes [9,10], particularly among those with higher tendencies to engage in social comparisons [11]. Comparisons with others who are perceived to be better off than oneself (upward comparisons) tend to have a more marked negative impact on well-being than comparisons with others who are perceived to be on an equal level (lateral comparisons) or who are perceived to be worse off than oneself (downward comparisons) [12]. Upward social comparisons have consistently been related to more negative self-judgments, lower self-esteem [9], and the presence of disordered eating behaviors [13,14]. The visual nature of many social media platforms creates a rich environment for upward social comparisons, particularly with respect to performance and physical appearance, which can have negative consequences for well-being [12,15,16]. For example, a meta-analysis of 156 studies found that social comparison is positively associated with body dissatisfaction, especially among women and younger people [17]. Furthermore, adolescents and young adults often portray ideal versions of themselves on social media using advanced filters, photo editing, and video editing. They may seek out experiences with specific considerations of how such content will be perceived when posted on social media, rather than how intrinsically motivating it is [18,19]. Young adults viewing this carefully curated content often perceive others on social media as having better lives [20] and thus are prone to the negative effects of these comparisons. Indeed, a systematic review and meta-analysis of 70 published studies showed that social comparison on social media is positively related to higher levels of depression and anxiety [21].

### Intervening on Social Comparisons

A growing body of work has investigated the impact of interventions designed to reduce social comparisons made during social media use. A subset of these interventions targets a lack of realism in social media content by manipulating participants’ exposure to different content. For instance, a study found that participants who primarily viewed selfies of people without makeup reported lower facial dissatisfaction than those who viewed selfies of people with makeup [22]. Likewise, participants who viewed parodied versions of celebrity images reported lower body dissatisfaction and greater positive mood than those who were exposed to thin idealized images of celebrities [23]. In another intervention, women who saw side-by-side images of idealized versus nonidealized targets reported lower body dissatisfaction than those who viewed the idealized images alone [24].

Although changing the social media content with which people engage can reduce the negative impact of social comparisons, such an approach may not be realistic. Moreover, not all upward social comparison is harmful. Unlike comparisons that foster contrast between oneself and others, comparisons that foster assimilation (ie, the belief that one can obtain the same status as someone else) are characterized by a selective focus on the similarities between oneself and others [25], which can motivate positive feelings about oneself [26].

Consistent with this evidence, other interventions have targeted how participants interact with potentially harmful social media content using, for example, self-compassion and mindfulness-based interventions. Self-compassion refers to kindness toward oneself, acceptance of one’s humanness, and understanding that negative experiences are universal [27]. Self-compassion may help reduce the negative impact of social media by fostering more stable self-worth and less externally contingent self-esteem. People with higher self-compassion tend to feel less inadequate and less judged or evaluated than people with lower reported self-compassion when exposed to the coveted experiences of others [27,28]. Mindfulness-based interventions enhance users’ attention to their present experiences and foster curiosity, rather than judgment, about one’s states. Mindfulness is negatively related to fear of missing out (FoMO), which refers to the apprehension that others may be having more positive experiences than oneself [29,30]. Similar to upward social comparisons that decrease positive affect, FoMO is pervasive on social media and related to more depressive symptoms [29]. Social media users who endorse higher mindfulness also report lower depression than users with lower mindfulness [31], and mindfulness-based interventions have shown promise in reducing body dissatisfaction and negative mood [32,33].

Although promising, other studies have found no evidence in support of these interventions designed to impact how people interact with social media [28,34]. These contradictory findings could stem from methodological differences, including intervention duration or the lack of control over prior states. It is also possible that these strategies are effective only among those with a higher tendency to compare themselves with others, as these people tend to be more negatively impacted by comparisons on social media [10,11,23,24,34].

### Savoring Interventions

A related strategy, yet less explored with reference to social media, is savoring. Savoring is an emotion regulation approach by which people focus their awareness on pleasant experiences and appreciate the pleasure of past, present, and future experiences [35,36]. At a trait level, savoring appears to moderate the relationship between positive or negative personal experiences and various outcomes, including happiness [37], life satisfaction [38], and depression [39,40]. At a state level, savoring can bolster and maintain levels of reward sensitivity [41] and improve people’s capacity to recognize and enjoy positive experiences even in the face of negative events [39].

Savoring is a promising target for psychological interventions [42]. A 6-week intervention that involved weekly sessions where savoring skills were taught reduced negative affect and increased positive affect in a sample of people newly diagnosed with HIV [43]. Similarly, a 2-week intervention showed that participants who engaged in and logged daily savoring practices reported lower levels of depressive symptoms and negative affect than those who did not receive the intervention [44]. Another study used social media platforms as a means to practice savoring by asking participants to describe and post about joyous activities performed during the week on social media platforms [45]. This intervention helped decrease depressive symptoms and negative mood compared with inactive controls.

Although a growing body of literature suggests that savoring can increase positive affect and reduce negative affect and depression, this skill has received little attention as a means to manage the negative impact of social comparisons on social media. In this project, we built on the concept of savoring and adapted it to be an interpersonal, empathic process we term “social savoring”: a focus on feeling happy or joyous for the positive experiences of another person. Social savoring attempts to enhance one’s positive feelings in response to *others*’ positive experiences, amplify attention to these positive feelings toward others [37], and thereby increase a sense of connection with others [35]. In essence, people focus on how good it feels to experience happiness and gratitude for another person when that person experiences a joyous moment. Empathy has been shown to be a powerful tool for enhancing the feelings of social connectedness and self-esteem [46]. When empathy is integrated with strategies that foster savoring, the resulting combination may prove to be a useful alternative to social comparisons.

### This Study

The purpose of this pilot study was to test the effectiveness and acceptance of a brief computer-delivered social savoring intervention to improve the quality of young adults’ interactions with social media, ultimately increasing social connectedness and self-esteem and reducing depressive symptoms, loneliness, and contingent self-worth. The present intervention introduced social savoring as a task in which participants were asked to observe (on social media) a joyous moment experienced by someone else, reflect on what it would feel like for that person to have that experience, and then allow themselves to feel joy for that other person. To better understand how the intervention impacted participants’ typical experiences with social media, we assessed their social media interactions for 7 days before receiving the intervention and 6 days after receiving the intervention.

We hypothesized that, relative to baseline, participants in the intervention group would report fewer depressive symptoms, lower loneliness, less contingent self-worth, reduced social comparison orientation, less FoMO, greater social connectedness, and higher self-esteem at posttest. At the daily level, we also expected that, on days after the intervention, participants in the intervention group would report decreased loneliness and social comparison and increased social connectedness, state self-esteem, and positive affect in response to social comparison compared with control participants.

## Methods

### Recruitment

#### Participants

Participants were 55 college students (mean age 19.29, SD 0.93 years; n=43,78% women and n=23, 42% White) enrolled in a private Southeastern university in the United States. Participants were recruited from the departmental research participation pool during the 2021 spring semester. Interested students completed a screening questionnaire to indicate where they currently resided and completed the Iowa-Netherlands Social Comparison Orientation Scale [47]. Those who were aged ≥18 years, lived in the United States, and scored >22 (out of 55) on the Social Comparison Orientation Scale—indicating a higher tendency to engage in social comparisons—were eligible to participate in the study.

#### Power

A 2-week, in-person, savoring intervention [44] in college students found an effect size of Cohen *d*=0.41 for the difference in the average level of depression between control and intervention participants. A post hoc power analysis indicated that this study had 85% power to find an effect size of Cohen *d*≥0.41 with an α of .05 and sample of 55 participants.

#### Data Exclusion

**Figure 1:**
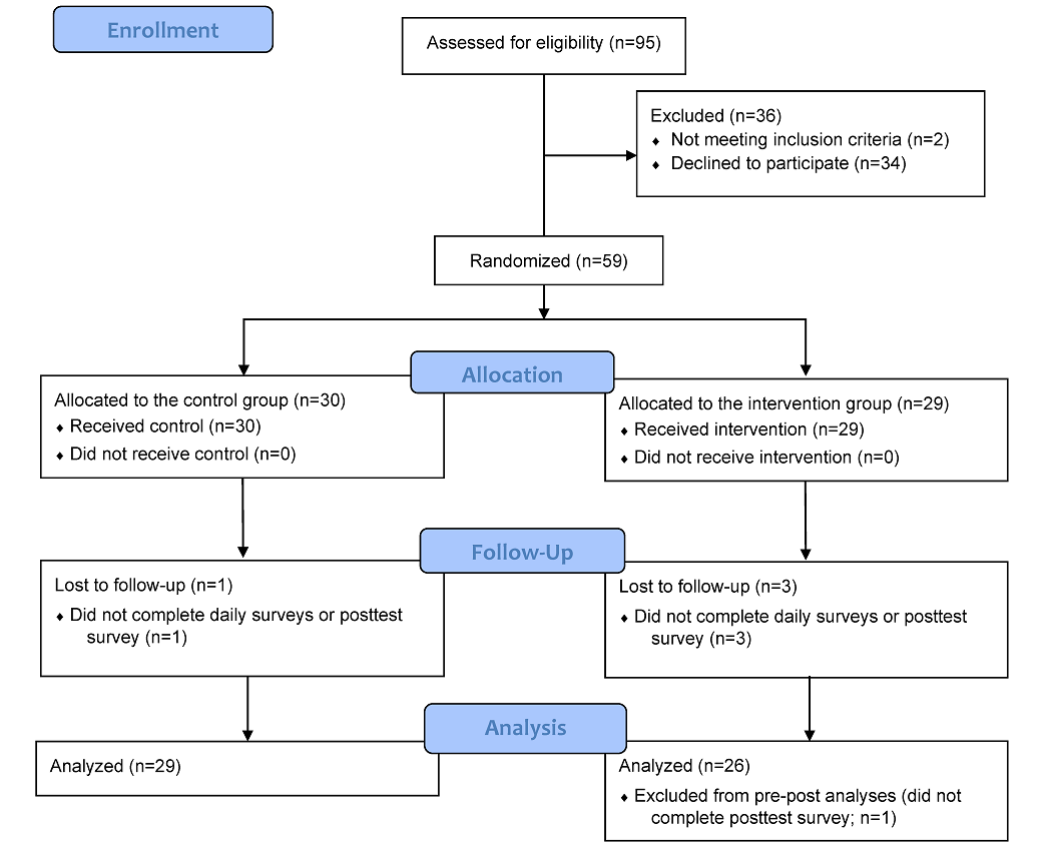
CONSORT (Consolidated Standards of Reporting Trials) flow diagram of trial.

As shown in Figure 1, of the 95 participants who completed the screening survey advertised on the web, 59 (62%) completed the study. A total of 7% (4/59) of participants completed only the baseline survey but did not complete any daily surveys or the posttest survey, and 2% (1/59) of participants from the intervention condition completed the daily surveys but did not complete the posttest survey. The latter participant was excluded from the analyses of pretest and posttest responses but was included in the analyses of daily interactions. Among the participants who completed at least 1 of the 13 daily surveys (55/59, 93%), compliance was high: participants completed an average of 12.45 (SD 1.09; range 8-13) surveys, resulting in a compliance rate of 96%.

### Ethics Approval

All procedures and materials were approved by the Duke University Campus Institutional Review Board (protocol 2021-0066) and are available on the web [48].

### Procedure

This study followed an intensive longitudinal design to enhance statistical power and deepen our understanding of participants’ typical interactions with social media. After providing consent to participate, participants completed the baseline survey (day 1) and were randomly assigned to a waitlist control or intervention condition. On the following day, all participants completed the first of 13 surveys (for days 2 to 14) delivered daily by text and email. After completing the seventh daily survey (day 8), participants who received the social media use intervention (26/55, 47%) viewed the intervention video and were instructed to practice the intervention skills daily (for approximately 5 minutes) and continue completing the daily surveys (days 9 to 14). Participants in the waitlist control condition (29/55, 53%) did not receive any new material but continued completing daily surveys through day 14. On the last day of the study (day 15), participants completed the posttest survey, which included items for assessing their experience with the intervention in addition to the same items as those in the baseline survey (day 1).

### Materials

#### Overview

Participants completed a baseline survey, daily surveys, and a posttest survey, as described in the subsequent sections. McDonald ω was used to calculate the internal consistency of the baseline and posttest scales. Participants in the intervention condition watched a video describing the social savoring intervention.

#### Baseline Survey

##### Social Comparison Orientation

Social comparison was assessed using the 11-item Iowa-Netherlands Comparison Orientation Measure [47], which assesses engagement in comparisons with others (eg, “I always like to know what others in a similar situation would do”). Responses were provided on a 5-point scale (1=*disagree strongly*; 5=*agree strongly*) and were summed so that higher values indicate higher social comparison. The internal consistency was appropriate for the present sample (ω=0.76).

##### Self-esteem

Self-esteem was measured with 20 items from the appearance (6 items, eg, “I feel unattractive”; ω=0.89), social (7 items, eg, “I feel that others respect and admire me”; ω=0.84), and performance (7 items, eg, “I feel like I’m not doing well”; ω=0.63) subscales of the State Self-Esteem Scale [49], which assesses the extent to which one feels positively valued in the moment. Responses were reported on a 5-point scale (1=*not at all*; 5=*extremely*) and were averaged so that higher values indicate higher levels of appearance, social, and performance self-esteem.

##### Depression

Depression was assessed using the 21-item Beck Depression Inventory–second edition (BDI-II) [50], which asks participants to report on various symptoms of depression (eg, self-criticalness and sadness) by selecting the descriptions that best reflect their experiences over the last 2 weeks, including the day of the survey (eg, “I cry over every little thing”; ω=0.87). Response options range from 0 (indicating no presence of a symptom) to 3 (indicating the extreme presence of a symptom). BDI-II scores were summed so that higher values indicate higher levels of depressive symptoms (possible range 0-63).

##### Trait Loneliness

Trait loneliness was assessed using the 20-item UCLA Loneliness Scale [51], which assesses the feelings of loneliness and social isolation (eg, “I am no longer close to anyone”; ω=0.95). Participants answered the items on a 4-point scale (1=*never*; 4=*always*). Scores were averaged so that higher values indicate higher levels of loneliness.

##### Social Connectedness

Social connectedness was assessed using the 8-item Social Connectedness Scale [52], which assesses connectedness and closeness to others (eg, “I don’t feel related to anyone”; ω=0.95). Responses were provided on a 6-point scale (1=*strongly disagree*; 6=*strongly agree*), and negatively worded items were rescored so that higher values indicate higher levels of social connectedness.

##### Contingencies of Self-worth

Contingencies of self-worth were assessed using the appearance and approval from others subscales of the Contingencies of Self-Worth Scale [53]. The measure assesses the extent to which one’s self-esteem depends on the validation of one’s appearance (“My sense of self-worth suffers whenever I think I don’t look good”; ω=0.81) and approval from others (eg, “I can’t respect myself if others don’t respect me”; ω=0.83). Responses were provided on a 7-point scale (1=*strongly disagree*; 7=*strongly agree*), and negatively worded items were rescored so that higher values indicate higher levels of each subscale.

##### FoMO Scale

We measured FoMO using the 10-item FoMO Scale [29], which assesses the fear that others may be having rewarding experiences that one is “missing out” on (eg, “I fear my friends have more rewarding experiences than me”; ω=0.83). The response options ranged from 1 (*not at all true of me*) to 5 (*extremely true of me*). Scores were averaged so that higher values indicate higher levels of FoMO.

#### Daily Interactions

##### Loneliness

Loneliness was assessed using a 1-item measure of state loneliness [54] (ie, “I feel lonely”), with response options ranging from 1 (*not at all*) to 5 (*very much*).

##### State Self-esteem

State self-esteem was assessed using 1 item asking, “As a whole, how do you feel about yourself right now?” [49]. Responses were provided using a dial ranging from 0 (*terrible*) to 100 (*terrific*).

##### Daily Social Comparison

Daily social comparison was measured using 1 item asking, “To what extent did you compare yourself to the social media content you viewed today?” It was rated on a scale of 1 (*not at all*) to 5 (*a great deal*). Participants who responded with ≥2 were further asked, “Considering how you were feeling about yourself before engaging with social media, how did comparing yourself to the social media content make you feel?” Responses were provided on a 5-point scale ranging from 1 (*much worse*) to 5 (*much better*).

##### Social Savoring

Social savoring was assessed with 1 item asking how often participants engaged in social savoring while using social media that day. Response options ranged from 1 (*0%-20% of the time on social media*) to 5 (*80%-100% of the time on social media*). Only participants assigned to the social savoring condition saw this item, which was presented on days 9 to 14 (ie, after the delivery of the intervention).

##### Social Media Use

Each day, participants reported the top 3 contents with which they interacted the most while using social media that day. Response options included fitness and sports, dieting and eating, food and cooking, political and societal issues, memes and comedy, beauty, nature and animals, art, and vacation.

#### Intervention

**Figure 2:**
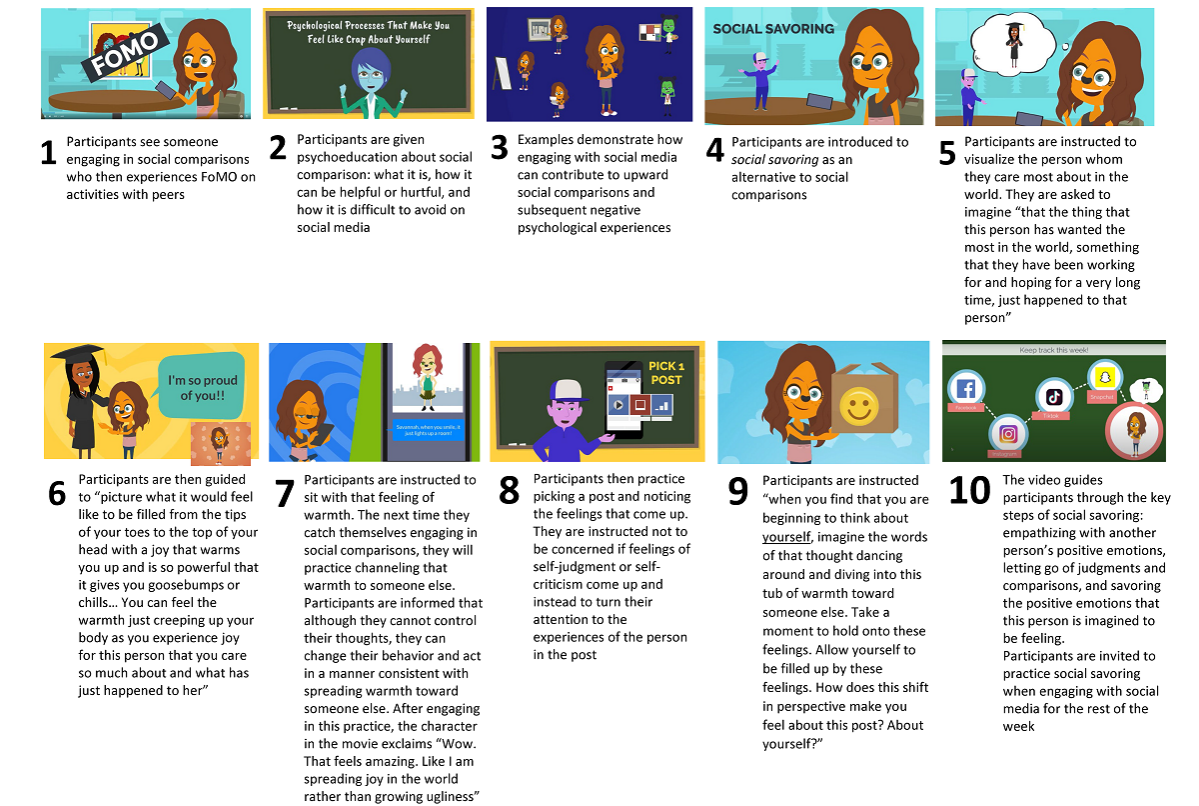
Summary of the intervention steps. FoMO: fear of missing out.

The intervention consisted of a 7-minute animated video presented on study day 8 to participants assigned to the intervention condition. The video introduced the concept of social comparison, explained how it manifests on social media and how social savoring can help minimize the impact of social comparisons on psychological well-being, and provided step-by-step instructions on how to practice social savoring (Figure 2). We intentionally adopted nonhuman characters in the video to reduce the possibility of physical appearance comparisons with the actors in the video. On subsequent study days (days 9 to 14), participants in the intervention condition were reminded of the basic steps of social savoring and asked to practice social savoring at least once before taking the daily survey.

#### Posttest Survey

On the final day of the study, participants completed the same battery of measures as those completed in the pretest phase: social comparison orientation (ω=0.79), appearance self-esteem (ω=83), social self-esteem (ω=0.87), performance self-esteem (ω=0.69), depression (ω=0.92), trait loneliness (ω=0.95), social connectedness (ω=0.95), appearance contingencies of self-worth (ω=0.77), approval from others contingencies of self-worth (ω=0.86), and FoMO (ω=0.91). Participants in the intervention condition also provided feedback on their experiences. Through open-ended questions, participants were asked whether social savoring was a new skill to them, what they thought about the cartoon-style intervention video, whether they agreed with the video’s message, how they perceived the duration of the intervention, and whether they had any suggestions for improvement. Through multiple-choice questions, participants were asked whether they would recommend this intervention to a friend, whether they preferred a live-action skit for the intervention video, and how likely they were to use this skill in the future.

### Statistical Analysis

This pilot study examined how social comparisons manifest on social media and whether social savoring can be a tool for helping buffer the negative impact of social comparisons. We performed multilevel analyses to examine whether the type of content participants daily engaged with was associated with the daily reports of social comparison and comparison affect. We used multilevel analyses to also examine whether the daily reports of social savoring were associated with the daily reports of social comparison, affect experienced from social comparison, loneliness, and state self-esteem.

To investigate participants’ acceptance of the intervention, we descriptively examined their responses to the multiple-choice and open-ended follow-up items, which were completed by participants in the intervention condition. To test the effectiveness of the brief computer-delivered social savoring intervention, we performed a 2-way repeated-measures ANOVA and paired-samples 2-tailed *t* tests to test for between-condition differences from baseline to posttest in depressive symptoms, loneliness, appearance- and approval from others–contingent self-worth, social connectedness, appearance, social and performance self-esteem, social comparison orientation, and FoMO. At the daily level, we used the same analytical approach to test the effect of the intervention on participants’ average daily levels of loneliness, social comparison, state self-esteem, and social comparison.

## Results

### Overview

As shown in Table 1, participants assigned to the control condition did not differ from those assigned to the intervention condition in any of the baseline measures.

**Table 1:** Equivalence between participants assigned to the control and intervention conditions.

Measure		Control (n=29)	Intervention (n=26)	Estimate^a^ *(df)*	*P* value
**Variable, mean (SD)**					
	SCO^b^	3.93 (0.08)	3.94 (0.11)	1.08 (51)	.29
	PSE^c^	3.32 (0.10)	3.21 (0.12)	0.38 (51)	.71
	SSE^d^	3.12 (0.17)	3.29 (0.20)	−0.87 (51)	.39
	ASE^e^	3.02 (0.13)	3.13 (0.18)	−0.32 (51)	.75
	Loneliness	2.08 (0.11)	1.92 (0.11)	1.06 (51)	.29
	Connection	3.65 (0.18)	4.04 (0.18)	−0.92 (51)	.36
	Appearance-contingent self-worth	4.96 (0.12)	4.90 (0.12)	0.68 (51)	.50
	Approval from others–contingent self-worth	3.41 (0.22)	3.48 (0.23)	−0.94 (51)	.35
	FoMO^f^	2.49 (0.14)	2.76 (0.15)	0.79 (51)	.31
	BDI-II^g^	11.54 (8.63)	11.32 (10.15)	−0.71 (51)	.48
**Demographics**					
	Women, n (%)	21 (72)	25 (96)	2.26 (4)	.13
	White, n (%)	19 (65)	17 (65)	3.73 (4)	.44
	Age (years), mean (SD)	19.47 (0.17)	19.10 (0.17)	1.52 (51)	.14

^a^Estimate refers to the between-participant 2-tailed *t* test coefficient for all scales and age and chi-square test coefficients for the women and White demographics.

^b^SCO: social comparison orientation.

^c^PSE: performance self-esteem.

^d^SSE: social self-esteem.

^e^ASE: appearance self-esteem.

^f^FoMO: fear of missing out.

^g^BDI-II: Beck Depression Inventory–second edition.

### Daily Reports of Comparison

The participants completed 685 daily surveys. Participants reported interacting the most with memes and comedy content in 56.5% (387/685) of all daily surveys, political and societal issues in 23.9% (164/685) of all daily surveys, fitness and sports content in 23.5% (161/685) of all daily surveys, beauty-related content in 21.8% (149/685) of all daily surveys, food and cooking content in 19.9% (136/685) of all daily surveys, dieting and eating content in 14.3% (98/685) of all daily surveys; and vacation (68/685, 9.9%), art (47/685, 6.9%), and nature and animals (46/685, 6.7%) content in less than 10% of all daily surveys. Of the 55 participants who completed at least 1 daily survey, 53 (96%) reported engaging in social comparison on social media at least once during the daily survey period. Comparisons were reported in 63.6% (436/685) of observations. When a comparison was reported, participants most commonly reported feeling “about the same” after the comparison (245/436, 56.2% observations), followed by feeling “much worse” or “worse” (173/436, 39.7% observations). Participants reported feeling “better” or “much better” in only 4.1% (18/436) of the comparisons.

Multilevel analyses tested how the type of content was associated with the daily reports of comparison and comparison-related affect, regardless of condition (Table 2). To enhance the interpretability of the comparison direction variable, and because of our specific interest in comparisons that worsen affect, we dichotomized the outcome as 1=comparisons that worsened affect and 0=comparisons that did not alter or improved affect. The analyses were limited to the types of content that were reported in at least 10% of the observations. On days in which participants reported engagement with each fitness content, diet content, and beauty content, they reported greater comparisons and greater odds of engaging in comparisons that worsened affect relative to comparisons that did not alter or improved affect (within-person associations; beauty: odds ratio [OR] 1.86, 95% CI 1.02-3.37; fitness: OR 1.92, 95% CI 1.06-3.50; diet: OR 3.69, 95% CI 1.60-8.49). Participants who engaged with more political content or more meme content than their peers were less likely to report engaging in comparisons that worsened affect relative to comparisons that did not alter or improved affect (between-person associations). No other within- or between-person associations between the type of content engaged with and social comparisons were statistically significant.

**Table 2:** Associations between the type of content and comparison^a^.

		Comparison			Comparison affect		
		*b*^b^ (SE; 95% CI)	*P* value	β^c^	*b* (SE; 95% CI)	*P* value	β
**Within-person association**							
	Fitness	*0.33 (0.13; 0.07 to 0.59)* ^d^	*.01*	*.18*	*0.66 (0.31; 0.06 to 1.25)*	*.03*	*.15*
	Diet	*0.73 (0.13; 0.47 to 0.98)*	*<.001*	*.32*	*1.31 (0.43; 0.47 to 2.01)*	*.002*	*.27*
	Food	0.01 (0.10; −0.18 to 0.20)	.93	.00	0.35 (0.28; −0.20 to 0.90)	.21	.08
	Politics	0.05 (0.11; −0.17 to 0.28)	.65	.03	−0.13 (0.37; −0.85 to 0.59)	.72	−.03
	Memes	0.14 (0.09; −0.04 to 0.32)	.13	.09	0.09 (0.29; −0.49 to 0.66)	.77	.02
	Beauty	*0.33 (0.10; 0.13 to 0.54)*	*.001*	*.18*	*0.62 (0.31; 0.02 to 1.22)*	*.04*	*.15*
**Between-person association**							
	Fitness	−0.32 (0.28; −0.86 to 0.23)	.26	−.20	−0.88 (0.66; −2.17 to 0.41)	.18	−.23
	Diet	0.00 (0.25; −0.50 to 0.49)	.99	.00	0.25 (0.77; −1.27 to 1.76)	.75	.05
	Food	0.11 (0.39; −0.65 to 0.86)	.79	.05	−1.11 (0.91; −2.89 to 0.67)	.22	−.21
	Politics	0.11 (0.26; −0.40 to 0.62)	.67	.07	−*2.02 (0.75; −3.48 to −0.56)*	*.01*	−*.55*
	Memes	−0.43 (0.22; −0.86 to 0.01)	.05	−.29	−*1.75 (0.66; −3.04 to −0.46)*	*.01*	−*.51*
	Beauty	−0.09 (0.27−0.63 to ; 0.45)	.74	−.05	0.48 (0.73; −0.95 to 1.91)	.51	.11

^a^The coefficients in the “comparison” column refer to the extent to which participants engaged in social comparisons by type of content. The coefficients in the “comparison affect” column correspond to the log odds that social comparisons were associated with negative affect relative to unchanged or positive affect. Negative coefficients reflect lower odds that participants felt more negative about themselves (relative to unchanged or more positive), and positive coefficients reflect greater odds that participants felt more negative about themselves (relative to unchanged or more positive).

^b^Unstandardized regression coefficient.

^c^Standardized regression coefficient.

^d^Significant results are shown in italics.

### Intervention Acceptability

Of those who completed the intervention and posttest survey (25/55, 45%), all but 4% (1/25) of participants answered at least 75% of the items about the intervention. In response to the multiple-choice items, most participants indicated that they were *likely* to practice social savoring in the future (18/25, 72%), that they would recommend the skill to others (*maybe/yes*: 23/25, 92%), and that they preferred our animated video over a live-action skit (22/25, 88%).

In response to the open-ended items, most participants reported that social savoring was a new skill to them (21/25, 84%) and that they agreed with the message of the video (24/25, 96%). Most participants (22/25, 88%) responded positively to the open-ended item that asked for thoughts on the video, including that they liked the intervention video (eg, “I liked the cartoon”), found the video helpful to convey information (eg, “It nicely summarized the concept in an entertaining way”), and found the video easy to understand (eg, “The cartoon was easy to understand”). A total of 4% (1/25) of participants reported that they thought that the cartoon was “a little cheese-y but got the point across,” and another 4% (1/25) indicated that they “thought it was weird that they weren’t all people and were like monster people.” When asked about the duration of the intervention, only 4% (1/23) of participants who responded to this open-ended question reported that it took longer than they would have liked, whereas the remaining participants (22/23, 96%) had generally positive reactions to the intervention duration, reporting—for example—that the intervention “did not take very long, [it was] very convenient” and “helped [them] reflect on [themselves] and [their] abilities” and that they “found [themselves] surprised that [they] could complete the skill so quickly and have it become normal practice.”

### Associations With the Reports of Social Savoring

On average (mean 2.01, SD 0.99; based on averaged response categories 1=0%-20%, 2=20%-40%, 3=40%-60%, 4=60%-80%, and 5=80%-100%), participants in the intervention condition reported engaging in social savoring for 20% to 40% of their time spent on social media during the intervention period. Nevertheless, there was variability in the extent to which participants reported engaging in social savoring such that participants indicated engaging in social savoring for 0% to 20% of their time in 45% of their reports, 20% to 40% of their time in 25% of their reports, 40% to 60% of their time in 16% of their reports, 60% to 80% of their time in 9% of their reports, and 80% to 100% of their time in 5% of their reports. Reports of social savoring increased in the days following the intervention (days 9 to 13; *b*=0.09, 95% CI 0.03-0.16, SE 0.03; *P*=.01), indicating greater social savoring engagement over time.

Multilevel models assessed whether the daily reports of social savoring were associated with the daily reports of social comparison, affect in response to social comparison, loneliness, and state self-esteem. Associations were not significant for social comparison (*P*=.82), social comparison affect (*P*=.30), and loneliness (*P*=.19). For state self-esteem, within-person (*b*=4.88, 95% CI 1.03-8.74, SE 1.97; *P=*.01) and between-person associations (*b=*6.70, 95% CI 1.48-11.93, SE 2.67; *P=*.01) emerged. The within-person associations indicate that on days in which participants reported engaging in more social savoring, they also reported greater state self-esteem (β=.18). The between-person associations indicate that participants who engaged in more social savoring relative to their peers also reported greater state self-esteem (β=.41).

### Effect of the Intervention on Pre-Post Assessments

We found main effects of time of assessment on social comparison and connectedness (refer to Table 3 for means): at posttest, participants reported lower levels of social comparison in the control (*t*_27_=2.33; *P*=.01; η_p_^2^=1.00) and intervention (*t*_24_=3.51; *P=*.002; η_p_^2^=0.34) conditions and higher levels of connectedness in the control (*t*_27_=−6.85; *P*<.001; η_p_^2^=0.63) and intervention (*t*_24_=−3.99; *P*=.001; η_p_^2^=0.40) conditions.

**Figure 3:**
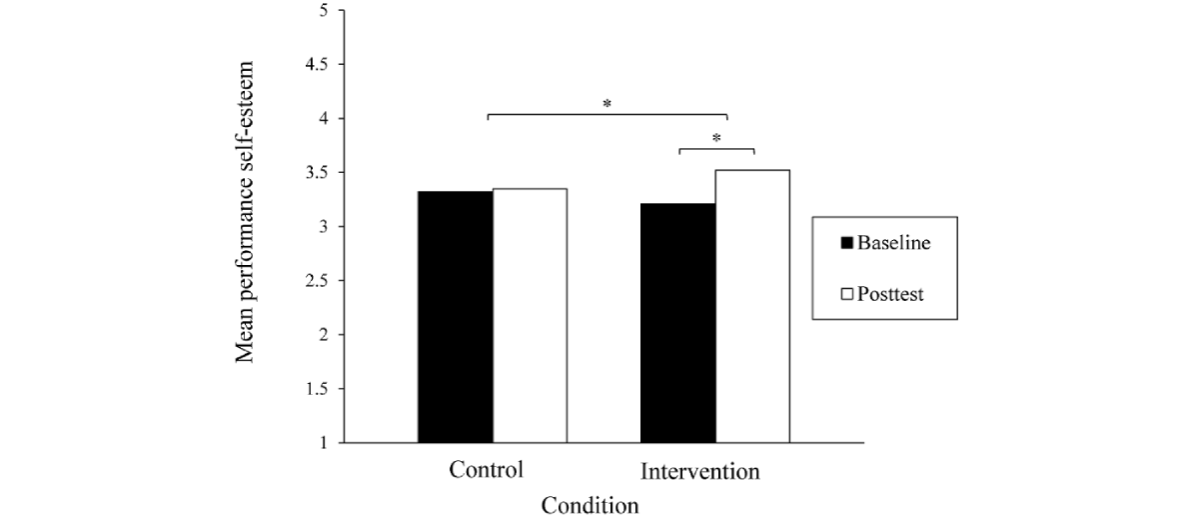
Mean performance self-esteem by time and condition. Higher scores indicate higher levels of performance self-esteem. Significant differences are marked with an asterisk.

Two-way mixed ANOVAs (Table 3) indicated that there was a significant effect of the interaction between condition and time on the level of performance self-esteem. As shown in Figure 3, participants in the intervention condition reported higher levels of performance self-esteem at posttest than at baseline (*t*_24_=−3.61; *P*=.001; η_p_^2^=0.35), whereas the levels of performance self-esteem among those in the control condition did not differ across time points (*t*_27_=−0.42; *P*=.68; η_p_^2^=0.01). Although participants in the intervention condition also reported higher levels of appearance self-esteem at posttest than at baseline (*t*_24_=−2.83; *P*=.01; η_p_^2^=0.25) and those in the control condition did not (*t*_27_=−0.55; *P*=.59; η_p_^2^=0.01), the interaction between condition and time indicated that the between-group difference was not significant.

**Table 3:** Differences in the pretest and posttest scores on key variables.

Variable	Control, mean (SD)			Intervention, mean (SD)			Time × condition	
	Pretest	Posttest	Pretest		Posttest	Estimate		*P* value
SCO^a^	3.93 (0.41)	3.72 (0.53)	3.94 (0.54)		3.62 (0.52)	0.72		.40
PSE^b^	*3.32 (0.54)* ^c^	*3.35 (0.50)*	*3.21 (0.58)*		*3.52 (0.43)*	*6.19*		*.02*
SSE^d^	3.12 (0.90)	3.15 (0.88)	3.29 (0.99)		3.42 (0.84)	0.40		.53
ASE^e^	3.02 (0.71)	3.07 (0.77)	3.13 (0.89)		3.41 (0.69)	3.62		.08
Loneliness	2.08 (0.57)	1.92 (0.56)	1.92 (0.53)		1.88 (0.44)	0.21		.65
Connection	3.65 (0.98)	4.19 (1.03)	4.04 (0.89)		4.48 (0.92)	0.51		.48
Appearance-contingent self-worth	4.96 (0.66)	4.96 (0.71)	4.90 (0.62)		4.88 (0.49)	0.01		.92
Approval of others–contingent self-worth	3.41 (1.15)	3.52 (1.19)	3.49 (1.14)		3.51 (1.35)	0.14		.71
FoMO^f^	2.49 (0.75)	2.42 (0.88)	2.76 (0.76)		2.64 (0.89)	0.09		.77
BDI-II^g^	11.54 (8.63)	11.32 (10.15)	12.72 (7.92)		11.08 (7.35)	0.89		.35

^a^SCO: social comparison orientation.

^b^PSE: performance self-esteem.

^c^Significant results are shown in italics.

^d^SSE: social self-esteem.

^e^ASE: appearance self-esteem.

^f^FoMO: fear of missing out.

^g^BDI-II: Beck Depression Inventory–second edition.

### Effect of the Intervention on Daily Interactions With Social Media

To examine the effect of the intervention on participants’ daily experiences with social media, we separately averaged their responses to the items on loneliness, social comparison, state self-esteem, and the impact of social comparison on days 2 to 8 (ie, before the introduction of the intervention) and days 9 to 14 (ie, after the introduction of the intervention). Thus, we created 2 composite scores for each daily variable: one referent to the days before the intervention and another referent to the days after the intervention. We then performed 2-way within-participant ANOVAs to test whether participants in the intervention condition differed from those in the control condition in their average daily experiences on days 2 to 8 and days 9 to 14.

As shown in Table 4, participants who watched the intervention video did not report any changes in loneliness (*P*=.24), state self-esteem (*P*=.92), comparisons on social media (*P*=.82), or feelings associated with comparisons (*P*=.76).

**Table 4:** Effects of the intervention on the daily reports of loneliness, self-esteem, social comparison, and comparison affect.

		State loneliness	State self-esteem	Social media comparison	Comparison affect^a^
**Control**					
	Pretest, mean^b^ (SD)	28.28 (21.26)	65.98 (15.33)	2.12 (0.64)	2.73 (0.47)
	Posttest, mean^c^ (SD)	23.85 (22.07)	65.84 (20.72)	2.01 (0.70)	2.69 (0.39)
	*F* test (*df*)	1.88 (1)	0.14 (1)	1.54 (1)	0.27 (1)
	*P* value	.18	.71	.23	.61
**Intervention**					
	Pretest, mean (SD)	29.34 (20.30)	66.14 (15.17)	1.87 (0.48)	2.59 (0.38)
	Posttest, mean (SD)	25.75 (17.96)	65.88 (18.00)	1.84 (0.61)	2.62 (0.37)
	*F* test (*df*)	1.45 (1)	0.01 (1)	0.50 (1)	0.10 (1)
	*P* value	.24	.92	.82	.76
**Condition × time**					
	*F* test (*df*)	0.36 (1,53)	0.11 (1,53)	0.31 (1,53)	0.34 (1,50)
	*P* value	.85	.74	.58	.56
	η^2^	0.00	0.00	0.01	0.01

^a^“Comparison affect” in this table refers to how positively participants felt about themselves after engaging in social comparisons; higher scores indicate feeling more positively, and lower scores indicate feeling more negatively.

^b^Pretest mean=average score on days 2 to 8 of the study.

^c^Posttest mean=average score on days 9 to 14 of the study.

## Discussion

### Principal Findings and Comparison With Prior Work

#### Overview

This pilot study assessed the acceptability, feasibility, and effectiveness of a novel social savoring intervention for reducing potentially harmful interactions with social media. Most participants reported positive perceptions of the web-based intervention and that they were likely to use the social savoring skill in the future. We found evidence of the effectiveness of the intervention in key psychosocial outcomes. Overall, we provide foundational research for future investigations on using social savoring to mitigate the negative effects of social media use.

#### Social Savoring Intervention

Our results show that this novel social savoring intervention is both acceptable and feasible. Participants generally reported a positive perception of the social savoring intervention in response to direct questions about their attitudes toward the intervention. That participants sustained high compliance with daily activities and increased their daily use of the (largely novel) social savoring skill after its introduction is further evidence of the acceptability and feasibility of the intervention. In addition, most participants expressed an interest in using the social savoring skill in the future and indicated that they would likely recommend this skill to a friend.

Our data provide preliminary evidence of the effectiveness of this intervention in promoting mental health. We found evidence of both between- and within-person associations between social savoring and self-esteem: participants who reported more social savoring than their peers also reported higher state self-esteem (between-person association), and participants who reported more social savoring than their own average on a given day reported higher state self-esteem on that day (within-person association). As participants reported savoring and self-esteem only once per day, the directionality of these associations is unclear. Social savoring involves focusing on pleasant experiences [35,36], and those who feel more positively about themselves may have an easier time engaging in this practice [55]. By contrast, savoring has previously been found to boost positive affect [56]. Future research using multiple same-day assessments can elucidate the directionality of these associations. Importantly, results from the posttest provide some support that the intervention was effective in increasing self-esteem in that performance self-esteem increased from pretest to posttest for the intervention group but not for the control group. The significant association in this domain, and not the others, could be an artifact of our sample of undergraduates coming from a highly selective university, making the intervention particularly effective in fostering the savoring of competence-related accomplishments. Alternatively, this finding may indicate that engaging in social savoring enhances positive self-evaluations via the prosocial nature of the activity.

Participants in both the control and intervention groups reported reduced social comparison at posttest compared with baseline. As demonstrated in prior research, asking participants to reflect and report on their psychological states can increase self-awareness and cause behavior changes [57]. The possible increases in self-monitoring in the control group may have inadvertently muted intervention effects.

Engaging in social savoring was associated with daily self-esteem but not with the reports of general social comparison, social comparison affect, loneliness, or depression. The lack of association with social comparison could indicate that social savoring does not necessarily reduce comparisons in general. Additional practice with social savoring may be necessary to shift people’s initial tendency from engaging in a comparison to savoring the positive feelings of another person’s experience. Alternatively, reducing the frequency of comparisons may not be necessary to buffer the negative effects of social comparisons. In fact, the potentially positive effects of social comparisons (eg, feelings of optimism, admiration, and inspiration) are consistent with the goals of social savoring [37].

Importantly, savoring was not associated with the consequences of social comparisons in that engaging in more savoring was unrelated to how participants felt about themselves after comparisons with social media content. This is consistent with our expectation that savoring operates differently from social comparisons because savoring shifts participants’ focus to the positive experiences of others rather than to what those experiences mean for themselves. The lack of association between savoring and loneliness could be because social media content is not exclusively socially oriented; for example, it can be appearance oriented [16] or career oriented [15]. Thus, effects may have been absent when there was a mismatch between the target of savoring and the measured outcome. Future research should incorporate daily measures of well-being that are specific to various domains to assess potential differences.

Contrary to prior work [41], we did not find an association between social savoring and depression. However, this finding may be a consequence of a misalignment between the wording of the BDI-II and the duration of the study. Specifically, the BDI-II items asked participants to think about their experiences over the previous 2 weeks [50], which included both the time before and after learning the social savoring skill for those in the intervention condition. Although participants in the intervention condition descriptively reported lower levels of depression at posttest compared with baseline, it is possible that the perceived change was dampened by the inclusion of psychological experiences that occurred before the intervention took place.

Consistent with the evidence that social media is visually oriented and evokes appearance comparisons [16], participants in the intervention group showed higher appearance self-esteem in the posttest than in the pretest, whereas participants in the control condition did not; however, the difference between the 2 groups was not statistically significant. Given the finding that appearance-oriented content was especially likely to evoke comparisons and comparisons that worsened affect, it is possible that our sample size was too small to detect between-group differences in the effect of the intervention on participants’ perceptions of the highly curated and attractive visual content of others. Future studies with larger samples may be able to detect this difference if present.

Finally, the absence of baseline-to-posttest changes may be attributable to the fact that some measured domains tend to be stable, trait-level characteristics. Social comparison orientation, contingencies of self-worth, and FoMO may simply be too stable for a short-term intervention to evoke meaningful changes [53]. Our lack of effects could be because participants need to build their social savoring skill over time to impact trait-level characteristics or because our participants geared social savoring toward specific domains, such as responses to appearance-oriented content. Future research assessing longer-term social savoring practice and investigating more specific domains (eg, appearance esteem) is needed to test these conjectures.

#### Comparison Experiences

Nearly all participants engaged in social comparison on social media at least once daily during the data collection period. Participants most commonly reported that comparisons did not change their affect (245/436, 56.2%); comparisons that worsened affect were reported in 39.7% (173/436) of the observations, and comparisons that improved affect were quite rare (18/436, 4.1%). Although past research has confirmed that comparisons that improve affect are rare on social media, research has also found that comparisons that worsen affect are more common than those that do not alter it [12]. Our finding of less frequent comparisons that worsen affect relative to comparisons that do not alter affect could be owing to differences in methodology: whereas past research [12] has exclusively assessed appearance comparisons, we examined comparisons more generally. Past research with adolescents indicates that people tend to highlight their physical appearance in their social media posts [58], which may inflate the likelihood of engaging in comparisons that worsen affect, specifically in the appearance domain [59]. Because we assessed a range of comparison domains (beyond just appearance), we would expect to observe a comparatively lower number of comparisons that worsen affect. Furthermore, our results suggest that college students may be less likely to engage in comparisons that worsen affect in other domains, such as food and politics.

Participants reported most often engaging with meme content, although fitness and sports, food, political and societal issues, and beauty content were also reported in a notable minority of observations. Regardless of the condition, on days in which participants engaged with fitness, diet, and beauty content, they reported a greater likelihood of engaging in comparisons and comparisons that worsen affect. These types of content are unified in that they are appearance oriented, suggesting that these types of content may be particularly potent in facilitating upward appearance comparisons. Appearance comparisons that worsen affect may be especially harmful to well-being [12], raising concerns about how these comparisons affect the in-the-moment well-being of social media users. Importantly, these associations were not observed at the between-person level, indicating that participants who engaged with more appearance-related content, in general, did not necessarily also engage in more comparisons or report worse affect in response to comparisons. Nonetheless, future longitudinal research can better ascertain how these appearance-related comparisons accumulate in the relationship between these comparisons and well-being over time.

Participants who engaged with more politics- and meme-related content than their peers reported a lower likelihood of engaging in comparisons that worsened affect (and, therefore, a greater likelihood of experiencing comparisons that did not change or improved affect). It is possible that these types of content portray others’ misfortunes, which could enhance the behavioral tendency to perceive oneself as better off than others. The findings of this study highlight a need to adopt a nuanced perspective that integrates various disciplines in studying how comparisons unfold for different types of content. Although viewing certain types of content may be beneficial in one domain (eg, memes associated with fewer comparisons that worsen affect), engagement with these types of content may be maladaptive in other domains (eg, perpetuating stereotypes).

### Limitations and Future Directions

The findings of this study are limited to a small sample of primarily White and women college students enrolled in a selective private Southeastern university in the United States. We attempted to replicate these findings in a sample of community members recruited through social media sites (eg, Instagram [Meta Platforms Inc], Reddit [Reddit Inc], Facebook [Meta Platforms Inc], and Twitter [Twitter Inc]). However, recruitment through these platforms was limited, and it was difficult to gain sufficient interest and retention from the community members. Indeed, of the 574 community members who completed the baseline survey, 48 (8.4%) provided good quality data (ie, no “straight-lining,” no nonsense responses to open-ended items, and no spam or repeated IP addresses), and only 11 (1.9%) completed the daily surveys to sufficient extent, a limited response rate that precluded comparisons with the present findings. These findings suggest the need for the development of innovative retention strategies if this intervention is to be implemented on a larger scale.

In addition, factors outside the content of the intervention appear to have influenced participants’ self-reported experiences. Specifically, the evidence that loneliness and social comparison levels decreased in both intervention and control groups suggests that merely reporting their own social media use daily influenced participants’ perceptions of these domains—and potentially how they interacted with social media. Another limitation is that we were unable to verify the true extent to which participants practiced the skills. Future studies using interventions such as the one used in this study may benefit from including a written practice during the introduction to the intervention, as done in the study by Hurley and Kwon [44], and daily written exercises to verify that participants spent time practicing social savoring.

Finally, the changes brought about by the COVID-19 pandemic may have shifted how participants interacted with social media such that social media use may have generally supported social connections. Given the restrictions imposed on participants at the time of the survey (eg, primarily web-based classes and restrictions on the size of gatherings), FoMO may have been less common than it would have been before the pandemic and thus less flexible to change.

Despite these limitations, the initial evidence from this pilot study suggests that a web-based social savoring intervention may help minimize the potentially harmful consequences of social media use. A fruitful avenue for future work is to examine the effectiveness and acceptance of this intervention for people diagnosed with disorders characterized by higher levels of comparison with others (eg, eating disorders). Specifically, savoring the joys of others may be a helpful strategy to feel more positively about one’s own skills and physical appearance.

Future studies should examine the effectiveness, feasibility, and acceptance of this intervention in a larger and more diverse sample. Given the evidence of an association between social comparisons on social media and eating disorders, depression, and anxiety [16,21,54,60], it would be valuable to examine the effectiveness of the present intervention among those with such psychopathologies. With changes such as those described earlier (eg, adding a daily written practice), a web-based intervention may be more easily delivered and accessed by a wider portion of the population.

### Conclusions

In summary, a 7-minute animated intervention video that taught the skill of social savoring improved self-evaluation in performance domains. Participants valued this prosocial approach to engaging with social media content, as evidenced by the increased practice of this skill over time and their purported willingness to share this technique with friends. Such promising results with such minimal intervention suggest that the use of social savoring as more tightly integrated with social media engagement (eg, cued by certain social media content, behaviors, and platforms) may result in a more potent invention capable of enhancing prosocial feelings and social connections while mitigating the harm of social comparisons.

## Abbreviations

BDI-II: Beck Depression Inventory–second edition
FoMO: fear of missing out
OR: odds ratio
